# Suffocation in an Infant, from Retraction of the Base of the Tongue

**Published:** 1846-12

**Authors:** 


*Suffocation in an Infant, from Retraction of the Base of the Tongue, connected with Defect of the Frænum.*—Dr. Fairbairn relates two interesting cases of this description, in the *Northern Journal of Medicine:* in the first, he states that the face presented a peculiar conformation; the superior part projected forwards, and gave a sharp appearance to the countenance; while the lower part was much depressed, the chin presenting a small flattened surface, instead of the rounded and projecting natural form; the expression of the countenance was similar to that observed in individuals who have had the body of the lower jaw excised. From a case somewhat similar, which occurred to him some years before, he suspected that there was malformation about the throat; and, on looking into the mouth, detected a fissure in the soft palate, which allowed the posterior nares and the vomer to be seen; the alveolar processes of the lower jaw were opposed to the back part of the hard palate above; and the tongue, which appeared short, and thick at its root, lay posterior to the palate, the apex alone projecting. On dropping a little sugar and water into the mouth cautiously, it was readily swallowed; but, whenever it was given in any quantity, it got into the nares, and produced much irritation, with cough and a sense of suffocation. A tea-spoonful of castor oil was administered on the Tuesday, with a little sugar and water, which acted upon the bowels. Early on Wednesday morning an attempt was made to apply her to the breast, but it was found that she could not suck. Shortly after this, her breathing became oppressed and irregular, with occasional heavy sighing. On his visit in the forenoon, he was particularly struck with the change; and, on examining the body of the child, he found that the face and extremities had assumed a purplish tint; the heart was beating irregularly and tumultuously; the little patient lay in a state of profound sleep, from which she could not be roused. He then supposed that there must be some malformation of the heart to account for the change which had supervened. The child died at two o'clock, P. M., on the 8th of May, without any apparent struggle. Some time before death, a little sack-whey was dropped into the mouth, but she could not swallow.

*Sectio Cadaveris.*—On opening the mouth, the posterior nares, the posterior border of the vomer, the upper wall of the pharynx, and the inferior apertures of the Eustachian tubes were readily seen; the anterior surface of the lower jaw lay posterior to the hard palate in the closed condition of the mouth; the soft palate was almost entirely deficient; the tongue was short and thick, retracted into the cavity of the pharynx, its convex dorsal surface resting upon the posterior wall of that cavity, and its base pressed upon the epiglottis and arytenoid cartilages, so as to completely obstruct the entrance of air into the larynx; the apex alone could be seen on looking into the mouth, while the jaw was forcibly depressed. Towards the apex, the margins were rolled inwards and upwards, so that the anterior part presented a deep furrow superiorly. The frænum appeared to be wanting, or was so slightly developed as not to bind down the lower surface of the tongue to the usual amount. The lower jaw was nearly flattened, forming a small segment of a circle, with a greater diameter than that of which a naturally formed jaw is a segment. The following measurements were made of this jaw, and of a normally formed jaw of the same period.

Breadth between the angles, including the thickness of the bone on both sides in a natural jaw, . . . . . . . . . 2 inches.
Breadth between the angles, including the thickness of the bone on both sides in a deformed jaw, . . . . . . . $2\frac{3}{8}$ "
Depth of the arch, including thickness of bone in a natural jaw, $1\frac{1}{2}$ "
Depth of the arch, bone not included, . . . . . . 1 1-10 "
Depth of the arch, including the bone in deformed jaw, . $\frac{3}{4}$ "
Depth of the arch, bone excluded in deformed jaw, . . . 6-10 "

The rami were somewhat smaller and less oblique; there was no malformation of the heart or other viscus; but the thoracic viscera presented the usual appearances in those dying from asphyxia.

In the second case, the face of the child presented a peculiar conformation; the upper part was prominent, while the region of the chin was much depressed; this arose from the flattening of the lower jaw, the upper projecting considerably beyond it. A little sugar and water was dropped into the mouth, which was swallowed, but when given in any quantity it caused considerable irritation, sometimes passing out at the nostrils, producing sneezing, coughing, and a sense of suffocation. On looking into the mouth, a cleft was seen in the soft palate with a bifid uvula; the posterior borders of the palate bones formed an acute angle in the mesial line. From this malformation it was found that it could not suck. He directed that he should be nourished by dropping into his mouth, cautiously, milk procured from a wet nurse. The milk thus procured was rather old, and being diluted with water, did not nourish the child. His bowels became irregular, and he had an attack of convulsions; these ceased on getting his bowels into a better condition with the use of the warm bath and cold applications to his head. He suggested the trial of ass's milk, which was procured and regularly given him; he continued to thrive well, and became a fine healthy child.

About the third month, he began to suck the thumb of the left hand, the palmar surface being carefully applied to the deficiency of space in the soft palate, whilst the fore and middle fingers were applied to the left side of the nose. This showed a very remarkable and striking provision of nature, by which this defect was remedied, and he was enabled to take in his nourishment and swallow with perfect safety, without any feeling of irritation in the nostrils, or sense of choking. His food was given him with a spoon, or more frequently with a bottle furnished with a teat, which was conveyed by the side of the thumb into the mouth, and allowed to be sucked in. When he felt hungry, he never failed to apply his thumb and commence sucking. During the whole period of infancy, great care was necessary in the preparation of his food; it consisted of ass's milk, smooth gruel, arrow root, and other farinaceous substances reduced to a pulp. He continued in this way until he began to feed himself, about his second year, when the use of the thumb was discontinued; however, if at any time he was incautious in taking too much food into his mouth, or swallowing hurriedly, instantly a sense of choking would supervene, the face would get livid, and, to all appearance, he would seem as on the eve of being attacked by convulsions. After birth he was not permitted to get out into the open air for six months; he suffered much from snifters, being particularly liable to attacks of cold from the slightest exposure even within doors, and had a constant discharge of saliva from the mouth. He is now five years of age; there is a considerable defect in his speech, and what he says has a decided nasal tone. The family know every word he says, unless he speaks rapidly. His faculties are equal if not superior to those of any boy of his age; he sings well, and is highly musical.

Remarks.—The cases related present a striking similarity in their general features, while the results proved very different. This may be accounted for from the malformation exhibiting nearly the same characters in both cases, with the exception of the inferior maxilla of the one being less depressed, and somewhat more angular than the other, and also from the defective condition of the frænum, so that the tongue was retracted, and the upper part of its root covered the rima glottidis, which obstructed the passage of the air and caused death. In surgical operations about the throat, accidents of the kind are apt to happen; great care is therefore necessary, should there be any threatening of the accident, to secure the tongue by ligature to some adjacent part. Were a similar case, observes Dr. Fairbairn, ever to occur to me again, I would never hesitate to transfix the apex of the tongue, and to attach it by ligature to the gum of the lower jaw.

